# Extracellular Vesicles from Apoptotic Cells Promote TGFβ Production in Macrophages and Suppress Experimental Colitis

**DOI:** 10.1038/s41598-019-42063-7

**Published:** 2019-04-10

**Authors:** Hua Chen, Shimpei Kasagi, Cheryl Chia, Dunfang Zhang, Eric Tu, Ruiqing Wu, Peter Zanvit, Nathan Goldberg, Wenwen Jin, WanJun Chen

**Affiliations:** Mucosal Immunology Section, NIDCR, NIH, Bethesda, MD 20892 USA

## Abstract

The clearance of apoptotic cells is an essential process to maintain homeostasis of immune system, which is regulated by immunoregulatory cytokines such as TGFβ. We show here that Extracellular Vesicles (EVs) were highly released from apoptotic cells, and contributed to macrophage production of TGFβ *in vitro* and *in vivo*. We further elucidated mechanistically that phosphatidylserine in EVs was a key triggering-factor, and transcription factor FOXO3 was a critical mediator for apoptotic EV-induced TGFβ in macrophages. Importantly, we found that macrophages pre-exposed to EVs exhibited an anti-inflammatory phenotype. More strikingly, administration of EVs *in vivo* promotes Tregs differentiation and suppresses Th1 cell response, and ameliorates experimental colitis. Thus, apoptotic-EV-based treatment might be a promising therapeutic approach for human autoimmune disease.

## Introduction

The clearance of apoptotic cells is an essential process to maintain homeostasis^1^. Phagocytes, including macrophages and immature dendritic cells, release immunoregulatory cytokines such as TGFβ, IL-10 and PGE2 during this process^2–5^, and these regulatory cytokines prevent and suppress activation of immune cells, and consequently maintain immune homeostasis. Among the known cytokines and factors, TGFβ, is highly released by macrophages upon the contact, engulfment and digestion of apoptotic cells^6^. TGFβ is a potent immunoregulatory cytokine that induces regulatory T cell, Th17 and Th9 cell differentiation, inhibits Th1, Th2 differentiation, and suppresses activation of B cells, macrophages, and dendritic cells^7–9^. We have previously shown a promising approach to treat autoimmune disease by inducing antigen-specific regulatory T cells *in vivo* through apoptotic cell-driven release of TGFβ by macrophages together with specific autoantigen peptide administration^10^.

Despite the recognition of the importance of apoptotic cell-driven TGFβ by macrophages in inducing and maintaining immune tolerance and homeostasis, the exact mechanisms by which apoptotic cells-stimulated macrophages produce TGFβ are incompletely understood^11^. Phosphatidylserine (PS), a molecule highly expressed on the membrane of apoptotic cells, is the key in initiating phagocytosis. It has also been reported that PS is an important molecule triggering the release of immune-regulatory cytokines in macrophages^6^. However, the receptors for phosphatidylserine on macrophages remain elusive. CD36 and TAM (Tyrosine Kinase Mer) receptor, which have been suggested to be PS receptors and associated with phagocytosis, were proposed as the receptors of the signaling pathway mediating TGFβ production, but this is still controversial^1,12^.

During the process of apoptosis, cells undergo extensive macromolecule changes such as cleavage and translocation^13^. Among them, the release of extracellular vesicles (EVs) is recently identified. EVs are membrane-bound structures released by cells, which are heterogeneous and generally classified into three groups: exosomes, microvesicles and apoptotic bodies^14,15^. EVs were previously considered as “cellular garbage”. However, accumulating evidence suggest that EVs are important mediators of intercellular communication^16–18^. For example, exosomes derived from IL-10-treated dendritic cells suppress inflammation and experimental arthritis^16^.

Release of EVs is observed in virtually all cell types, and additionally, apoptosis as well as proinflammatory cytokines promote the release of vesicles. Exosomes are the smallest multivesicular bodies-derived vesicles that sized 30–150 nm in diameter^15,19^. In view of this, we hypothesized that the mechanism of apoptotic cell-triggered TGFβ production by macrophages might involve the release of EVs from the apoptotic cells. Indeed, we show here that apoptotic cells released an increased quantity of EVs, and these EVs promoted macrophage to produce large amount of TGFβ. We further demonstrated mechanistically that transcription factor FOXO3 was involved in apoptotic-exosome-triggered TGFβ production in macrophages. Importantly, we found that the macrophages pre-exposed to EVs revealed an anti-inflammatory phenotype. More strikingly, we showed that EVs treatment suppressed Th1 cell proliferation *in vivo* and prevented gut inflammation in a mouse model of colitis.

## Results

### Apoptotic cells release more EVs than viable cells

**Figure 1:**
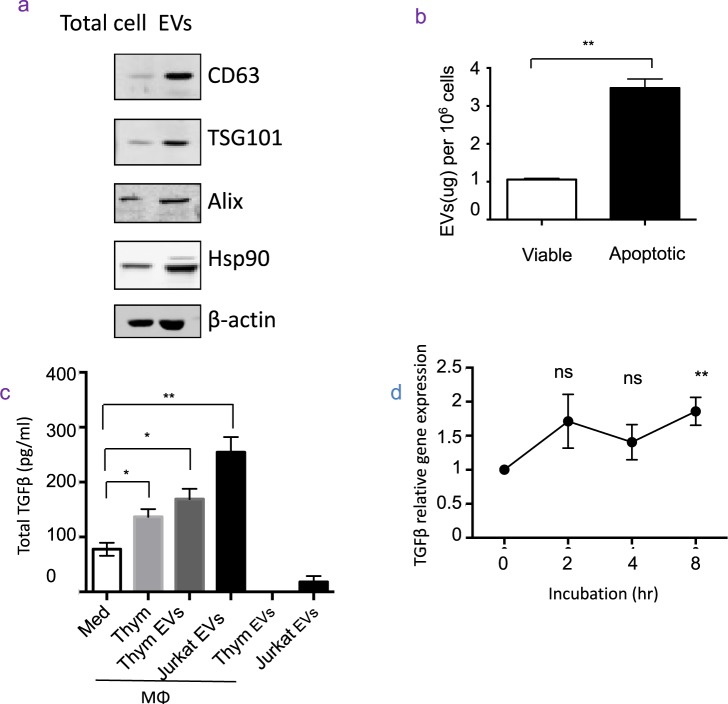
Apoptotic cell-derived EVs promote TGFβ in macrophages *in vitro*. (**a**) Western blotting of EVs markers: Total cell lysate (Total cell) and EVs of apoptotic thymocytes are immune-blotted with antibodies against CD63, TGS101, Alix, Hsp90 and β-actin, as indicated. Data were representative for two independent experiments. (**b**) EVs quantification: viable cells (n = 3) and apoptotic cells (n = 3) were cultured in serum-free medium for 6 hr, and EVs were extracted from the supernatant and quantified with BCA assay. Data were representative for two independent experiments. (**c**) TGFβ production of macrophages: peritoneal macrophages were cultured in serum-free medium (Med, n = 3) or stimulated with 0.5 × 10^6^ apoptotic thymocytes (Thym, n = 3), EVs derived from 40 × 10^6^ Thymocytes (Thym Exo, n = 3), or EVs derived from 20 × 10^6^ Jurkat cell (Jurkat Exo, n = 3) for 24 hr. The total TGFβ levels in supernatants were quantified by ELISA. Data were representative for three independent experiments. (**d**) TGFβ mRNA in macrophages: peritoneal macrophages (n = 4) were stimulated with EVs for 0, 2, 4 and 8 hr. RNA was then extracted and quantified for TGFβ1 and GAPDH mRNA by qPCR. Data of three independent experiments were combined.

We first isolated and characterized EVs from apoptotic cells. As shown in Fig. 1a, the characteristic markers of EVs, including CD63, TSG101, Alix and HSP 90, were enriched in EVs fraction, compared with total cell lysates. Electron microscopy and dynamic light scatter revealed the EVs derived from apoptotic and viable cells was 50–100 nm and 50–200 nm in diameter, respectively (Suppl Fig. 1A,B), which were consistent with exosomes. We then utilized mouse thymocytes as a model to quantify the proteins of EVs released from apoptotic and viable cells. Indeed, we found that the quantity of EVs measured by protein level from apoptotic cells were significantly larger than that from viable cells (Fig. 1b, Suppl Fig. 1C). Thus, apoptotic cells release more EVs than viable cells.

### Apoptotic cell-derived EVs promote TGFβ production in macrophages *in vitro*

We next determined the role of apoptotic cell-derived EVs in TGFβ production by macrophages. We isolated peritoneal macrophages from wild type C57BL/6 mice and cultured them with either apoptotic thymocytes or EVs isolated from apoptotic C57BL/6 thymocytes or Jurkat cell line (Fig. 1c). As expected, macrophages produced basal level of TGFβ at rest, and produced increased levels of TGFβ when exposed to apoptotic thymocytes^2,4^. Both EVs from apoptotic thymocytes and Jurkat cell line induced significantly higher amounts of TGFβ in macrophages (Fig. 1c), while EVs from apoptotic cells alone showed hardly any detectable TGFβ (Fig. 1c). We also used EVs derived from viable cells or necrotic cells to challenge macrophages, and found viable-cell-derived EVs also stimulated macrophages to produce TGFβ, although to a lesser extent, however, necrotic-cell-derived EVs did not promote TGFβ production (Suppl Fig. 1E). To confirm that the TGFβ elevation was due to increased transcription of TGFβ gene, we measured TGFβ1 mRNA levels in the macrophages upon exposure to EVs. Indeed, the TGFβ1 mRNA was gradually increased after EVs challenge, and reached statistical significance at 8 hrs after stimulation (Fig. 1d). Collectively, we show that EVs, while not a source of TGFβ themselves, are able to stimulate macrophage to produce increased levels of TGFβ *in vitro*.

### Apoptotic cell-derived EVs promoted TGFβ production macrophages *in vivo*

**Figure 2:**
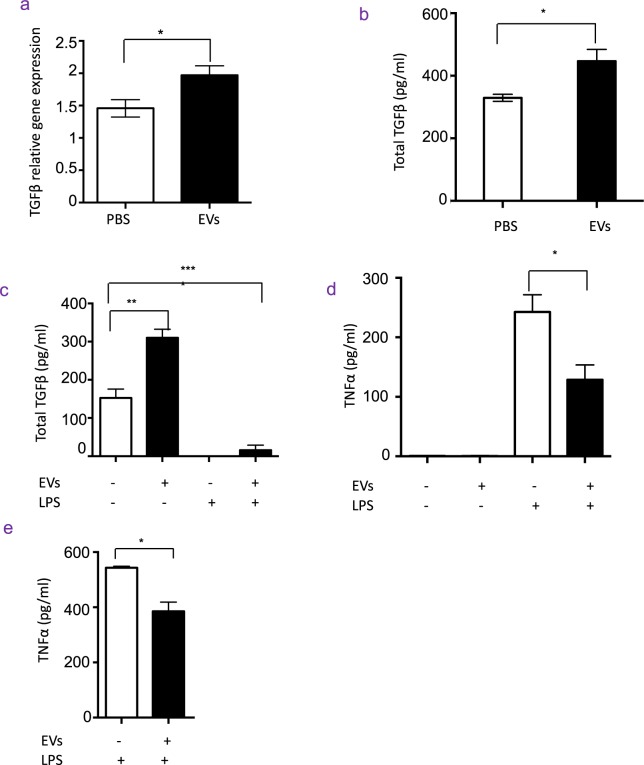
EVs promote TGFβ production in macrophages *in vivo*. (**a**) mRNA level of TGFβ expressed by macrophages *in vivo*: EVs (n = 4) or PBS (n = 4) were intraperitoneally injected into C57BL/6 mice, and peritoneal macrophages were harvested at 24 hr and quantified TGFβ and GAPDH mRNA. Data of two independent experiments were combined. (**b**) Protein level of TGFβ produced by macrophages *in vivo*: EVs (n = 3) or PBS (n = 3) were intraperitoneally injected to wild type C57BL/6 mice, and peritoneal macrophages were harvested at 24 hr and further cultured in serum-free medium for next 24 hr. The TGFβ in supernatant was quantified by ELISA. Data were representative for three independent experiments. (**c**) TGFβ production in macrophages challenged with LPS: EVs (n = 3) or PBS (n = 3) were intraperitoneally injected into C57BL/6 mice, and then challenged intraperitoneally with LPS (200 μg per mouse) 4 hr later. Peritoneal macrophages were harvested at 16 hr and further cultured in serum-free medium for next 24 hr. TGFβ in supernatant were quantified by ELISA. Data were representative for two independent experiments. (**d**) Plasma levels of TNFα in mice challenged with LPS: EVs (n = 3) or PBS (n = 3) was intraperitoneally injected into C57BL/6 mice, and mice were challenged intraperitoneally with LPS 4 hr later. Plasma was harvested at 16 hr and quantified for TNFα by ELISA. Data were representative for two independent experiments. (**e**) TNFα production in macrophages challenged with LPS: EVs (n = 3) or PBS (n = 3) were intraperitoneally injected into C57BL/6 mice, then challenged with intraperitoneal injection of LPS 4 hr later. The peritoneal macrophages were harvested at 16 hr and restimulated with LPS in serum-free medium for next 24 hr. The level of TNFα in supernatant was quantified by ELISA. Data were representative for two independent experiments.

To study whether EVs promote macrophages to produce TGFβ *in vivo*, we intraperitoneally injected apoptotic thymocyte-derived EVs into normal mice and harvested the peritoneal macrophages 24 hrs later. We observed that macrophages from mice that were injected with EVs exhibited higher levels of TGFβ1 mRNA (Fig. 2a) and TGFβ1 protein (Fig. 2b), compared with macrophages from mice injected with PBS. Thus, apoptotic cell-derived EVs induce TGFβ production in macrophages *in vivo*.

To investigate whether the macrophages pre-exposed to apoptotic EVs showed alteration of activation of macrophages *in vivo*, we injected EVs into C57BL/6 mice, then challenged them with i.p. injection of LPS 4 hours later to induce TNFα that is a pro-inflammatory cytokine and also a marker for macrophage activation. As expected, untreated macrophages exhibited basal levels of TGFβ (Fig. 2c), whereas injection of EVs significantly enhanced TGFβ production without induction of TNFα (Fig. 2c,d). Intriguingly, injection of LPS into mice significantly attenuated the TGFβ production of macrophages to undetectable levels of protein (Fig. 2c); pre-exposure to EVs partially rescued TGFβ protein production in macrophages reduced by LPS *in vivo* (Fig. 2c). We then examined the circulating levels of TNFα in the serum in the same treated mice. As expected, the levels of serum TNFα were undetectable in mice pretreated with PBS or EVs and LPS injection induced large amounts of TNFα in the blood (Fig. 2d). However, pre-administration of EVs into mice significantly decreased the levels of circulating TNFα induced by LPS (Fig. 2d). The decrease in circulating TNFα was indeed due to reduction of macrophage TNFα production, as TNFα secretion in macrophages isolated from peritoneal cavity of mice pretreated with EVs followed by LPS challenge was significantly reduced compared to macrophages from mice challenged by LPS alone (Fig. 2e). Collectively, the data indicates that EVs could promote TGFβ production in macrophages *in vivo*, as well as suppress their activation *in vivo*.

### Phosphatidylserine and FOXO3 are involved in apoptotic EV-triggered TGFβ in macrophages

**Figure 3:**
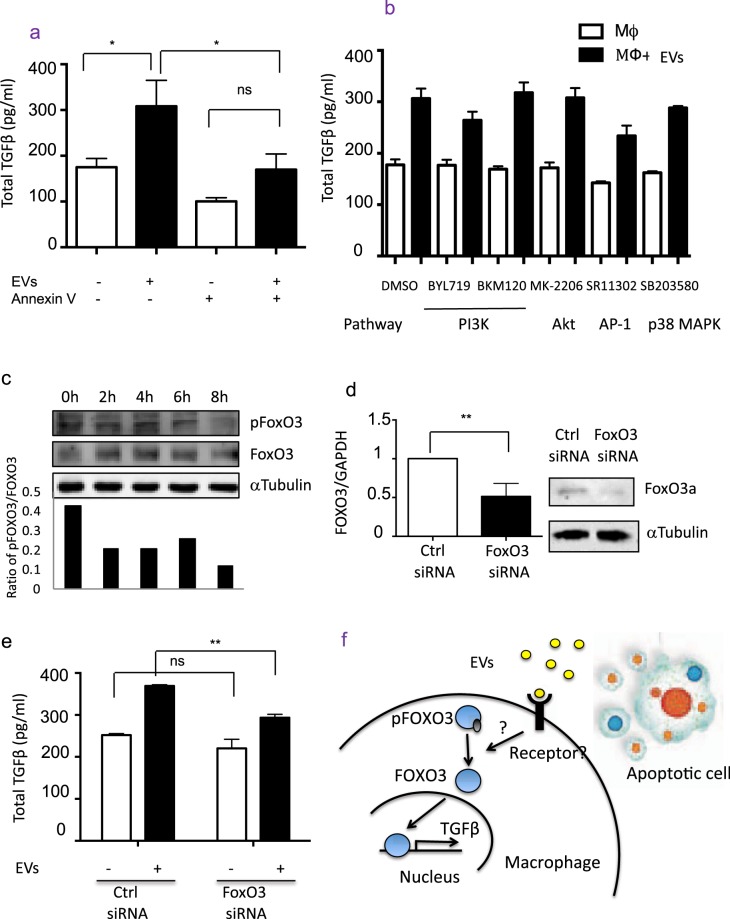
The mechanisms of TGFβ production in macrophages stimulated with EVs. (**a**) Annexin V inhibits the TGFβ production in macrophages stimulated by EVs: macrophages were pretreated with Annexin V (n = 5) or medium (n = 5) for 1 hr, then stimulated with or without EVs for 24 hr. The TGFβ was quantified in supernatant by ELISA. Data of five independent experiments were combined. (**b**) PI3K, AP-1 and p38 MAPK pathways are not involved: macrophages were pretreated with PI3K (BYL719 1 µM, BKM120 1 µM), Akt (MK-2206 1 µM), AP-1 (SR11302 1 µM) and p38 MAPK (SB203580 10 µM) inhibitors for 1 hr, then stimulated with (MΦ + EVs, n = 3) or without EVs (MΦ, n = 3) for 24 hr. The TGFβ was quantified in supernatant by ELISA. Data were representative for two independent experiments. (**c**) The expression of FOXO3 and pFOXO3 in macrophages stimulated with EVs: macrophages were treated with EVs for 0, 2, 4, 6 and 8 hr, then harvested and immunoblotted with antibodies against FOXO3, pFOXO3 and αTubulin. Data were representative for two independent experiments. (**d**) FOXO3 Knockdown in macrophages: macrophages were transfected with FOXO3 siRNA (n = 4) or control siRNA (n = 4). The mRNA and protein level of knockdown efficiency was confirmed at 24 and 48 hr. Data of four independent experiments were combined. (**e**) FOXO3 is implicated in the mechanism: macrophages were transfected with FOXO3 siRNA (n = 4) or control siRNA (n = 4) for 24 hr, then treated with EVs for 24 hr. The TGFβ was quantified in supernatant by ELISA. Data of two independent experiments were combined. (**f**) Scheme of EV-driven TGFβ pathway in macrophages.

We next investigated the underlying molecular mechanisms by which EVs induce TGFβ production in macrophages. PS has been reported as a key molecule that triggers macrophage TGFβ production^6^, and PS is highly expressed in EVs (Suppl Fig. 1G). We then tested whether PS is responsible for the TGFβ production stimulated by EVs. By blocking PS with Annexin V^20^, the TGFβ production of macrophages stimulated by EVs were significantly reduced (Fig. 3a), suggesting an important role for PS in EV-mediated TGFβ production.

We then investigated the molecular pathways involved. We first examined the roles of PI3K/AKT, p38 MAPK pathways that have been reported to be involved in TGFβ production in macrophages^11^. However, blockade of PI3K, p38 MAPK activities with their specific inhibitors did not abrogate the increase in TGFβ in macrophages stimulated with EVs (Fig. 3b), suggesting that alternative pathway(s) are involved. In human monocytes, transcription factor Foxo3a has been shown to bind to the TGFβ promoter and silencing Foxo3a attenuates TGFβ production, which implicates Foxo3a potentially regulates TGFβ transcription^21^. We found that phosphorylated FOXO3 (pFOXO3), the inactive form of FOXO3 which is localized in cytoplasm, was decreased in macrophages stimulated with EVs (Fig. 3c). This suggests that more active FOXO3 were likely binding to the promoter region of TGFβ to promote its transcription^21^. To confirm this, we knocked down the FOXO3 gene in macrophages prior to exosome treatment (Fig. 3d). We show that reduction of FOXO3 in macrophages partially but significantly decreased TGFβ production in macrophages triggered by EVs (Fig. 3e). Thus, our data indicates that PS is a key molecule in EVs and it promotes TGFβ production in macrophage, which at least in part is mediated by FOXO3.

### EVs treatment ameliorates experimental colitis

**Figure 4:**
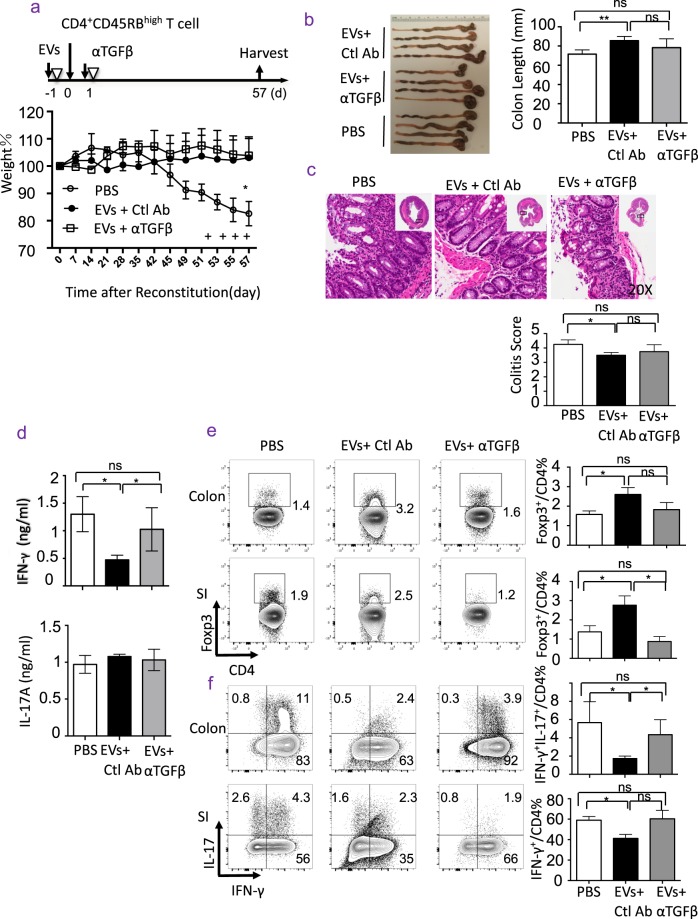
EVs treatment ameliorates colitis. (**a**) Disease course of colitis: Rag1^−/−^ mice were injected with PBS (PBS, n = 4), EVs in combined with control antibodies (EVs + Ctl Ab, n = 4), or EVs in combined with anti-TGFβ antibodies (EVs + αTGFβ, n = 4), prior to and after transfer of CD4^+^CD45RB^high^ T cells (300,000 cells). The body weight was monitored twice per week. Small intestine (SI), colon, mesenteric lymph node and spleen were harvested on day 57. Data were representative for three independent experiments. (**b**) The colon length of mice (n = 4). (**c**) Histological examination (H&E stain, X20 and X2 (insert)) (left) and colitis score (right) of colons. (**d**) Serum IFN-γ and IL-17 levels of mice (n = 4). (**e**) Representative flow cytometry (left) and frequency (right) of Tregs in lamina propria of colon and small intestine. (**f**) Representative flow cytometry (left) and frequency (right) of Th1 and Th17 cells in lamina propria of colon and small intestine.

The aforementioned findings that EVs trigger TGFβ production and prevent activation of macrophages encouraged us to investigate whether treatment with EVs was therapeutically applicable. We utilized a well-established mouse model of colitis in Rag1-deficient mice, which was induced by adoptive transfer of normal CD4^+^CD45RB^high^ T cells^22^. We treated mice with PBS, EVs alone, or in combination with anti-TGFβ neutralizing antibodies (See immunization scheme on Fig. 4a). As expected, the mice treated with PBS (untreated) developed severe diarrhea and weight loss (Fig. 4a, lower panel). Strikingly, mice treated with EVs maintained stable body weight (Fig. 4a). Intriguingly, mice treated with EVs and anti-TGFβ neutralizing antibody showed similar weight to mice treated with EVs alone (Fig. 4a). However, measurement of the colon length showed that EVs treatment prevented the reduction of the colon length caused by inflammation (Fig. 4b), and the colon length between mice treated with PBS and those treated with EVs plus anti-TGFβ antibody were comparable (Fig. 4b). Importantly, HE histology analysis of the colon revealed that the inflammatory cell infiltration in the colon was substantially decreased in mice treated with EVs (Fig. 4c, and data not shown), but no significant difference between mice treated with PBS or mice treated with EVs plus anti-TGFβ antibody were noted (Fig. 4c).

Analysis of the circulating levels of IFN-γ and IL-17, two known inflammatory cytokines involved in pathogenesis of colitis, revealed that EVs treatment significantly reduced serum levels of IFN-γ, but not IL-17 levels in the serum in mice (Fig. 4d). Neutralization of TGFβ with antibody almost completely reversed the decrease in serum IFN-γ levels in mice treated with EVs, although it did not have an effect on IL-17 levels in the serum (Fig. 4d). Finally, we examined IFN-γ- and IL-17-producing T cells in the gut, and showed consistently that EVs treatment substantially reduced IFN-γ^+^ IL-17^−^ (Th1) cells and IL-17^+^IFN-γ^+^ double-positive T cells, but not IFN-γ^−^IL-17^+^ (Th17) cells (Fig. 4f). Importantly, neutralization of TGFβ completely restored the frequencies of IFN-γ^+^ T cells in both colon and small intestine (Fig. 4f), but failed to significantly reverse the suppression of IL-17^+^ T cells, in mice treated with EVs (Fig. 4f). Moreover, EV-treated mice showed substantially increased frequency of Tregs both in colon and in small intestine compared to the untreated mice, and this increase in Tregs was completely abolished when anti-TGFβ antibody was injected with EVs treatment in mice (Fig. 4e). Collectively, we provide strong evidence that EVs treatment can ameliorate autoimmune colitis by increasing TGFβ production, which in turn promotes Treg induction but inhibits Th1 cell differentiation.

## Discussion

In this study, we determined that EVs released from apoptotic cells promoted macrophage production of TGFβ *in vitro* and *in vivo*. We further elucidated mechanistically that the phosphatidylserine in EVs was a key triggering factor, and transcription factor FOXO3 was a critical mediator for apoptotic EV-induced TGFβ in macrophages. Importantly, we showed that apoptotic cell-derived EVs suppressed experimental colitis and ameliorated the gut inflammation in CD4^+^CD45RB^hi^ adoptive transfer colitis model in mice by inhibiting IFN-γ^+^ inflammatory T cells and enhancing Tregs in a TGFβ-dependent manner.

EVs are released by virtually all types of cells^19^ and serv as a mechanism of cell-to-cell contact. We showed that the levels of EVs released from apoptotic cells were much higher than those released from viable cells, which is consistent with a previous report that apoptotic cells release more microparticles than viable cells^13^. Additionally, viable-cell-derived EVs also promoted macrophages to produce TGFβ, which suggested that the difference of quantity, rather than quality of the EVs, was responsible for promoting TGFβ production. However, it remains possible that the compositions of the apoptotic EVs and viable cell EVs might be different. Whether phagocytosis of apoptotic bodies is required for macrophages to release TGFβ is controversial. However, Chuang *et al*. demonstrated that cell-to-cell contact, but not necessarily phagocytosis, is sufficient for macrophages to release IL-10^23^, another key cytokine released during apoptosis. In light of this, our data suggest that more extracellular vehicles are released during apoptosis, and elevated EVs surrounding apoptotic cells might cause macrophages to “sense” and “react to” apoptotic cells.

Infusion of apoptotic cells represents a potential therapy for autoimmune conditions such as graft-versus-host disease, as this results in more Treg induction in a TGFβ-dependent manner through macrophages^24^. Apoptotic cells are easy to obtain, however, infusion of allogeneic whole cells has several limitations: first, the allogeneic cell may cause allergy; second, the apoptotic status of cells are not homogenous, which may contain necrotic cells and trigger “danger signals”; third, the batch-to-batch apoptosis rates of cells are variable, which makes titration of the optimal dose complex. As an alternative, we recently developed an approach to induce endogens apoptotic cells together with specific autoantigen administration to effectively treat autoimmune disease by inducing autoantigen-specific Tregs *in vivo*^10^. Here, we show that EVs derived from apoptotic cells have similar therapeutic effect as apoptotic cells. We showed that injection of EVs promoted TGFβ production by macrophages *in vivo*, and furthermore, ameliorated inflammation and promoted Treg differentiation. Our data suggest that EVs treatment significantly promotes TGFβ production by macrophages, deactivate macrophages, and alter T cell differentiation, which could be a potential approach for *in vivo* induction of Tregs to treat autoimmune diseases. Furthermore, given that EVs are much less complex than cells, the possibilities of allergy or triggering “danger signals” that may attenuate the immunoregulatory effect of apoptotic cells, are greatly reduced. Intriguingly, it was noted that TGFβ induced by EVs treatment seems to be required for the amelioration of the colitis, but not the prevention of the weight loss in this IBD model. In IBD model, IL-12 and IL-23 are responsible for weight loss and colitis, respectively^25^. However, in our study, we showed that EVs treatment mainly ameliorated aberrant Th1 cells but not Th17 cells in the gut. Therefore, the mechanisms underlying this differential regulation of systemic inflammation vs. gut colitis remain unknown and await for further investigation.

In our study, EVs derived from both mouse cells and human T cell line promote TGFβ production, which suggests that EVs from different species share common properties. During apoptosis, PS moves from the inner layer of cell membrane to the outer. During the releasing of EVs, the membrane asymmetry is lost and aminophospholipids including PS appear on the outer layer of EVs membrane^13,26^. By blocking PS, the effect of EVs was abolished, and this indicates the critical role of PS in EV-triggered TGFβ production in macrophages. For the downstream signal pathways, we determined that FOXO3 plays an important role, as reduction of FOXO3 by gene knockdown significantly down-regulates TGFβ production, consistent with an earlier finding that FOXO3 is a key transcription factor for TGFβ in human monocytes^21^. As the reduction of TGFβ in macrophages by FOXO3 knockdown was incomplete, it suggests that other molecular pathways are also involved.

ERK, p38 MAPK, PI3K and JNK pathways were reported to be involved in TGFβ production in a mouse fibroblast cell line, however, we did not find a role of these pathways in our study^11^. Thus, the complete and exact molecular pathways underlying EV-mediated TGFβ production in macrophages remains elusive and awaits further investigation.

In summary, we showed that apoptotic cells released significantly higher quantity of EVs that in turn promoted TGFβ production in macrophages, which is accomplished at least partially through PS-mediated pathway in a FOXO3-dependent manner. The EV-educated macrophages show an anti-inflammatory phenotype. Importantly, EVs administration *in vivo* promotes Tregs differentiation and suppresses Th1 cell response, and ameliorates experimental colitis. Apoptotic EV-based treatment might therefore be a potential therapeutic approach for human autoimmune diseases.

### Experimental Procedures

#### Mice

C57BL/6, Rag1^−/−^, DO11.10 and Balb/c mice were purchased from the Jackson’s Laboratory and maintained in the animal facility of National Institute of Dental and Craniofacial Research (NIDCR), National Institutes of Health (NIH). All mouse studies were performed according to NIH guidelines for use and care of live animals and approved by the Animal Care and Use Committee of the NIDCR. Experimental groups were 3 to 4 mice per group, done to be able to perform statistical analysis. Mice were randomly assigned to each group, but the experimenter was not blinded to group identity.

#### Cell line

Jurkat cells were maintained in the RPMI-1640 medium supplemented with Bovine serum (10%), L-Glutamine, Penicillin/Streptomycin.

#### Apoptotic cell induction

Primary murine thymocytes were irradiated with 30 Gy with a Gammacell 40 irradiator. Jurkat cells were UV-irradiated for 5 min.

#### EVs isolation and analysis

Thymocyte (40 × 10^6^/ml) and Jurkat cells (20 × 10^6^/ml) were induced apoptosis and cultured in serum-free X-VIVO medium for 6 hr, centrifuged at 400 g for 10 min to remove the cell pellet, followed by centrifuged at 5,000 g for 30 min to remove apoptotic bodies and large cell debris. EVs were collected by: (1) incubated with total exosomes isolation buffer (Life Technologies) at 4 °C overnight, and centrifuged at 10,000 g, 4 °C for 60 min; (2) centrifuged at 180,000 g for 2 hr, then washed with PBS and centrifuged again by ultracentrifugation^27^. EVs were stored at −80 °C until use. EVs were quantified using a BCA assay (Bio-Rad) and a CD63 ELISA (SBI System Bioscience). The size distribution of EVs was examined using dynamic light scatter (Malvern instruments). For flow cytometry analysis, EVs were incubated with beads coupled with anti-CD9 antibody (Life Technologies) and stained with Annexin V.

#### Macrophage isolation

Peritoneal lavage was incubated with anti-CD11b Microbeads (Miltenyi Biotec) and magnetically isolated as recommended by manufacturer. Purity was generally > 90% tested by flow cytometry.

#### TGFβ quantification

Cell culture samples were acid-activated and tested with ELISA kit according to the manufacturer’s instruction (Promega).

#### TGFβ RNA

RNA was extracted by RNeasy Mini Kit (Qiagen), reversed transcribed to cDNA (Life Technologies) and tested for TGFβ (Mm01178820_m1, Life Technologies) and GAPDH (Mm99999915_g1) mRNA by ABI 7500 real-time PCR (Life Technologies).

#### Colitis model

CD4^+^CD45RB^high^ T cells were FACS-sorted and injected into Rag1^−/−^ mice (300,000 cells per mouse). The mice were monitored with weight loss twice a week and were euthanized on day 57.

### Histological examination

Colon sections were fixed in 10% buffered formalin and embedded in paraffin, then cut, stained with hematoxylin and eosin, and examined. Histological grades were independently assessed in a blinded manner on a scale of 0–5, as follows: grade 0, no changes observed; grade 1, minimal scattered mucosal inflammatory cell infiltrates, with or without minimal epithelial hyperplasia; grade 2, mild scattered to diffuse inflammatory cell infiltrates, sometimes extending into the submucosa and associated with erosions, with minimal to mild epithelial hyperplasia and minimal to mild mucin depletion from goblet cells; grade 3, mild to moderate inflammatory cell infiltrates that were sometimes transmural, often associated with ulceration, with moderate epithelial hyperplasia and mucin depletion; grade 4, marked inflammatory cell infiltrates that were often transmural and associated with ulceration, with marked epithelial hyperplasia and mucin depletion; grade 5, marked transmural inflammation with severe ulceration and loss of intestinal glands.

#### Isolation of Lamina Propria Cells

The colon and small intestine of mice were harvested and intra-epithelial lymphocytes were removed. Tissues were digested with Liberase LT (Roche). Cells were stained for CD45, CD4, TCRβ, Zombie, IFN-γ, IL-17A and Foxp3 and analyzed using Fortessa LSR II (BD). Dead cells were excluded from analysis using Zombie Yellow Fixable Viability Kit (Biolegend).

#### Antibodies

The following antibodies were used for cell staining: anti-CD45 (30-F11), anti-CD4 (RM4-5), anti-CD8α (53–6.7), anti-CD25 (PC61.5), anti- DO11.10 (KJ1-26), anti- IFN-γ (XMG1.2), anti-IL17A (TC11-18 H10.1), and anti-Foxp3 (FJK-16s) from Biolegend. Anti-TGFβ antibody (1D11.16.8) and isotype control antibody (MOPC-21) were purchased from Bio X Cell.

#### Flow Cytometry

Cells were surface-stained with fluorochrome-conjugated, permeabilized (eBioscience), and then stained with antibodies against IFN-γ, IL-17A and Foxp3. Data were acquired on Fortessa LSR II (BD) and processed by FlowJo.

#### RNA silencing

Macrophages were incubated with INTERFERin (PolyPlus) and control or FOXO3 siRNAs (50 nM) for 24 hr. The knockdown efficiency was confirmed at 24 hr (mRNA level) and 48 hr (protein level).

#### Statistical Analysis

Data was pooled from two to three independent experiments and tested using Student’s *t*-test (unpaired two-tail). A *p*-value of < 0.05 was considered significant.

## Supplementary information

Suppl. Figure plus legends
