# Supplementary material for: Extracellular Vesicles from Apoptotic Cells Promote TGFβ Production in Macrophages and Suppress Experimental Colitis
Source: Sci Rep. 2019 Apr 10;9:5875. doi: 10.1038/s41598-019-42063-7 (PMC6458171; doi:10.1038/s41598-019-42063-7)
Supplement: Supplementary file 1 — Suppl. Figure plus legends [file 41598_2019_42063_MOESM1_ESM.pdf]

Extracellular Vesicles from Apoptotic Cells Promote TGFβ Production in Macrophages and Suppress Experimental Colitis

Hua Chen[1], Shimpei Kasagi[1], Cheryl Chia[1], Dunfang Zhang[1], Eric Tu[1], Ruiqing Wu[1], Peter Zanvit[1], Nathan Goldberg[1], Wenwen Jin[1], WanJun Chen[1*]

[1] Mucosal Immunology Section, NIDCR, NIH, Bethesda, MD 20892

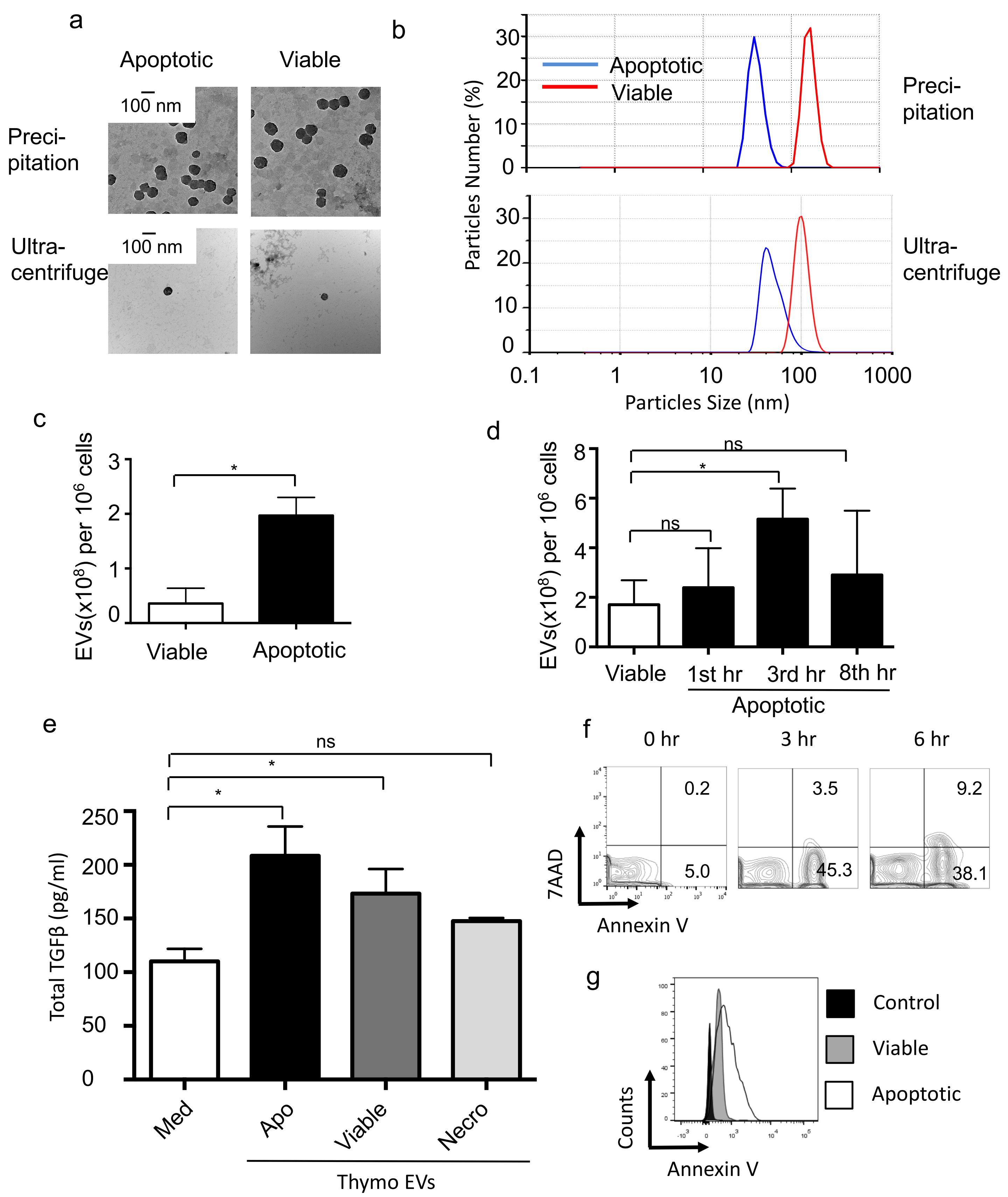

Suppl Figure 1

Supplemental Figure 1.

a. Electron Microscopy of EVs: EVs derived from apoptotic and viable Jurkat cells were purified by total exosomes isolation buffer (upper panel) or ultracentrifugation (lower panel). Electron Microscopy revealed copious amounts of particles with a diameter of 50-100 nm.

b. Dynamic light scatter of EVs: Purified EVs derived from apoptotic and viable Jurkat cells by total exosomes isolation buffer (upper panel) or ultracentrifugation (lower panel) were analyzed with dynamic light scatter (Malvern), giving a profile between 50-200nm in diameter.

c. EVs quantification: Apoptotic Jurkat cells (n=3) were cultured in serum-free medium for 6 hr. EVs were purified by ultracentrifugation, and were quantified using a CD63 ELISA kit, with a known concentration of CD63 as the standard curve.

d. EVs profile: Viable Jurkat cells were cultured for 1 hr (Viable), then were induced to apoptosis. The supernatant during the 1st hr (1st hr), 3rd hr (3rd hr), and 8th hr (8th hr) of apoptosis were harvested. EVs were purified by total exosomes isolation buffer and were quantified using the CD63 ELISA kit.

e. TGFβ production of macrophages stimulated with EVs derived from viable or necrotic cells: peritoneal macrophages were cultured in serum-free medium (Med, n=3) or stimulated with EVs isolated from $40x10^6$ apoptotic thymocytes (Apo, n=3), $40x10^6$ viable thymocytes (Viable, n=3), or $40x10^6$ necrotic thymocytes (Necro, n=3) for 24 hr.

f. Thymocytes apoptosis: Murine thymocytes were irradiated with 30 Gy with Gammacell 40 irradiator and cultured in cultured in serum-free medium for 0, 3 or 6 hr. Cells were stained with Annexin-V PE and 7AAD and evaluated apoptosis by flow cytometry.

g. PS expression on EVs: EVs from apoptotic (open) or viable (gray) jurkat cells were incubated with beads coupled with anti-CD9 antibody, stained with Annexin V or isotype control (black), and analyzed by flow cytometry.

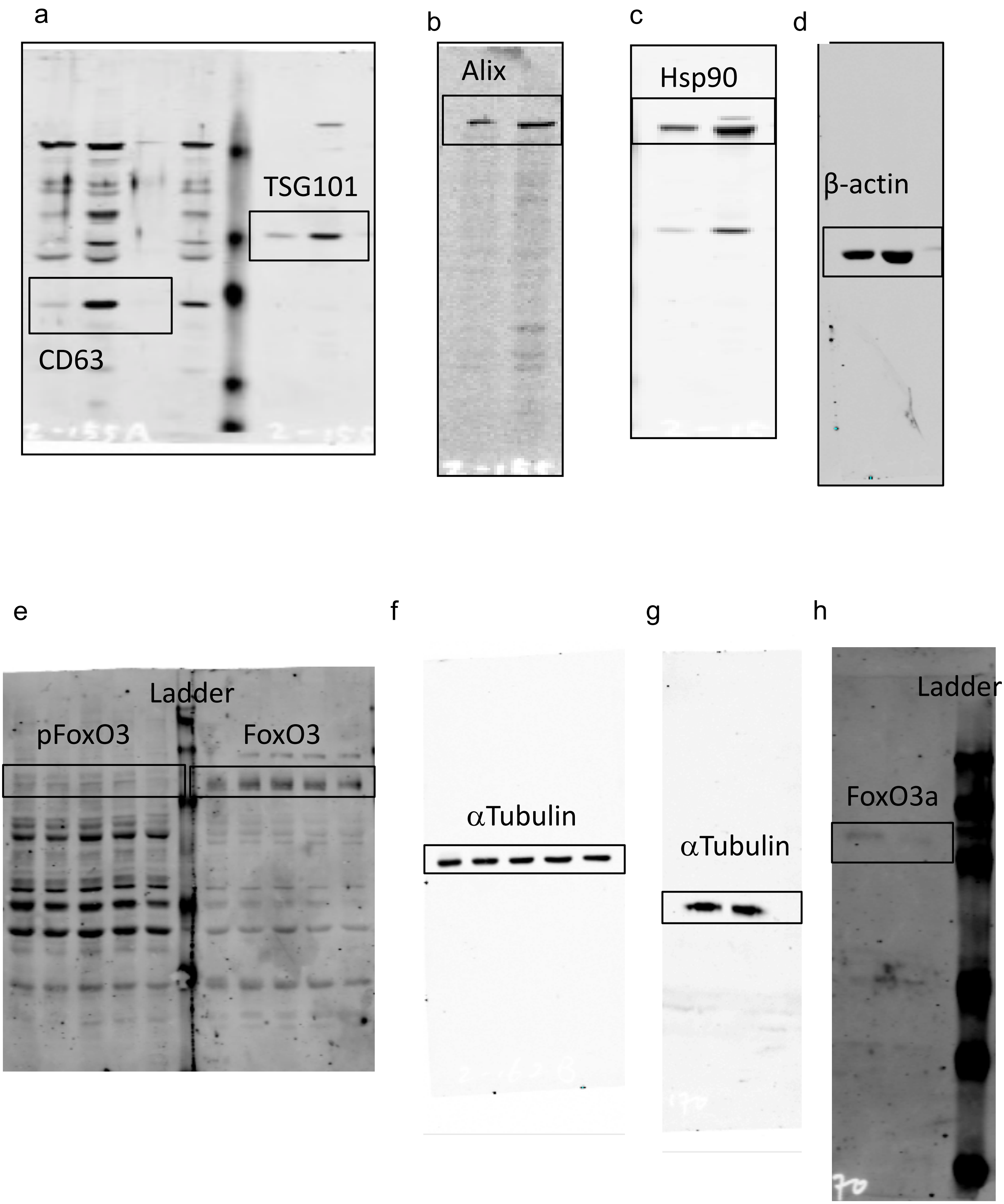

Suppl Figure 2

Supplemental Figure 2. Original Western Blotting Images.

a. Original western blotting of CD63 (left) and TSG101 (right) in Figure 1A.
b. Original western blotting of Alix in Figure 1A.
c. Original western blotting of Hsp90 in Figure 1A.
d. Original western blotting of β-actin in Figure 1A.
e. Original western blotting of pFoxO3 (left) and FoxO3 (right) in Figure 3C.
f. Original western blotting of αTubulin in Figure 3C.
g. Original western blotting of αTubulin in Figure 3D.
h. Original western blotting of FoxO3a in Figure 3D.
